# Gefitinib Analogue V1801 Induces Apoptosis of T790M EGFR-Harboring Lung Cancer Cells by Up-Regulation of the BH-3 Only Protein Noxa

**DOI:** 10.1371/journal.pone.0048748

**Published:** 2012-11-21

**Authors:** Bo Zhang, Jiao Jiao, Ying Liu, Liang-Xia Guo, Bo Zhou, Gang-Qin Li, Zhu-Jun Yao, Guang-Biao Zhou

**Affiliations:** 1 Division of Molecular Carcinogenesis and Targeted Therapy for Cancer, State Key Laboratory of Biomembrane and Membrane Biotechnology, Institute of Zoology, Chinese Academy of Sciences, Beijing, China; 2 State Key Laboratory of Bioorganic and Natural Products Chemistry, Shanghai Institute of Organic Chemistry, Chinese Academy of Sciences, Shanghai, China; 3 Graduate University of the Chinese Academy of Sciences, Beijing, China; 4 The Shenzhen Key Laboratory of Gene and Antibody Therapy, Division of Life and Health Sciences, Graduate School at Shenzhen, Tsinghua University, Shenzhen, China; University Magna Graecia, Italy

## Abstract

Treatment of non-small cell lung cancer (NSCLC) with drugs targeting the epidermal growth factor receptor (EGFR), e.g., gefitinib and erlotinib, will eventually fail because of the development of secondary mutations such as T790M in EGFR. Strategies to overcome this resistance are therefore an urgent need. In this study, we synthesized a dozen of novel gefitinib analogues and evaluated their effects on L858R/T790M-EGFR harboring NSCLC cells, and reported that one of these gefitinib mimetics, N-(2-bromo-5-(trifluoromethyl) phenyl)-6-methoxy-7-(3-(piperidin-1-yl)propoxy)quinazolin-4-amine (hereafter, V1801), triggered apoptosis of the NSCLC cells and overcame gefitinib-resistance in mice inoculated with NCI-H1975 cells. Though V1801 only moderately inhibited EGFR kinase activity, it markedly induced the expression of the BH3-only protein Noxa, and Noxa silencing significantly reduced V1801-induced apoptosis of NCI-H1975 cells. It is showed that V1801 interfered with the expression of the transcription factor c-Myc and the extracellular signal regulated kinase (Erk) pathway. V1801 in combination with proteasome inhibitor bortezomib exerted enhanced cytotoxicity in NCI-H1975 cells possibly due to potentiated induction of Noxa expression. These data indicate that gefinitib analogues with weak EGFR inhibitory activity may overcome drug-resistance via activation of BH-3 only pro-apoptotic proteins, and V1801 may have therapeutic potentials for NSCLC.

## Introduction

Lung cancer is the one of the most frequent neoplasm worldwide, comprising 17% of the total new cancer cases and 23% of the total cancer deaths in males [1]. Treatment of lung cancer is determined by the histologic type and stage at diagnosis, and mainly includes surgery, platinum doublet therapy, radiation therapy and targeted therapy, with an only 15% of five-year overall survival rate for all stages combined [2]. The epidermal growth factor receptor (EGFR)-targeting agents including EGFR-specific antibodies and tyrosine kinase inhibitors (TKIs) such as gefitinib and erlotinib, benefit a proportion of patients especially those never-smoke and of Asian origin [3]–[5]. However, treatment with gefitinib and erlotinib will eventually fail because of the development of acquired drug resistance resulting from amplification of the MET proto-oncogene or the threonine-to-methionine amino acid substitution at position 790 (T790M) of EGFR, which is detected in 50% of clinically resistant patients [6], [7]. The T790M mutation in EGFR affects the gatekeeper residue in the catalytic domain of the kinase that weakens the interaction of the inhibitors with the target [6]. Recent studies show that T790M mutation is a “generic” resistance mutation that will reduce the potency of any ATP-competitive kinase inhibitor [8]. In T790M EGFR-harboring cells, inhibition of EGFR by currently available second-generation EGFR-TKIs is not sufficient to physiologically prevent the emergence of cells that are still dependent on EGFR signaling [9]. Therefore, strategies to overcome EGFR TKI resistance remain practical needs in order to prolong survival time of patients with lung cancer.

Evading apoptosis is one of the hallmarks of cancer, and targeting apoptosis has become a cancer therapeutic strategy [10]. The critical apoptosis regulators, B cell CLL/lymphoma-2 (BCL-2) and its family proteins, can be divided into pro- and anti-apoptotic members, and the pro-apoptotic proteins can be subdivided into proapoptotic proteins and BH3-only subfamilies, based on their structural similarity of various Bcl-2 homology (BH) domains [11], [12]. Noxa is a BH3-only protein that is implicated in apoptosis associated with DNA damage, hypoxia or exposure to proteasome inhibitors [13]–[15]. Expression of Noxa in Hela cells strongly promotes cell apoptosis, while blocking the endogenous Noxa suppresses programmed cell death [16]. Accumulation of Noxa sensitizes apoptosis by binding to and neutralizing the antiapoptotic protein Mcl-1, leading to release and subsequent activation of Bax (BCL2-associated X protein) or BAK (BCL2-antagonist/killer) from Bax/Bak-Mcl-1 complex [13], [17], [18]. In addition to its role in apoptosis, Noxa also regulates diverse cellular functions in autophagic cell death and metabolism [19], [20]. Apoptosis-associated Noxa activation is primarily achieved through transcriptional upregulation by p53, E2F1, HIF1α, c-Myc, ATF3, and extracellular signal regulated kinase (Erk) pathway [14]–[16], [21]–[23]. However, the identity of the critical BH3-only proteins and the mechanism of their action following treatment by diverse apoptotic stimuli remain to be fully resolved.

To screen for the agents to overcome gefitinib-resistance, we here synthesized a number of novel gefitinib structural analogues and tested their inhibitory effects on EGFR kinase activity and proliferation of NCI-H1975 cells [24] which harbor L858R/T790M-EGFR [6], [7], [25] and wild-type MET without genomic amplification [26] that are resistant to gefitinib and erlotinib. We found a gefitinib mimetic, N-(2-bromo-5-(trifluoromethyl)phenyl)-6-methoxy-7-(3- (piperidin-1-yl)propoxy)quinazolin-4-amine (hereafter, V1801), moderately inhibited EGFR kinase activity but significantly suppressed cell proliferation and induced apoptosis in T790M EGFR-harboring non-small cell lung cancer (NSCLC) cells in vitro and in vivo. We further showed that up-regulation of Noxa underlain V1801's activity on NSCLC cells.

## Materials and Methods

### Agents

The gefitinib analogues (Table 1) were synthesized by our chemistry group, and the *N*-(3-chloro-4-fluoro-phenyl)-7-methoxy-6-(3-morpholin-4-ylpropoxy) quinazolin-4-amine (gefitinib) was purchased from J&K Chemical Ltd. All the compounds were dissolved in dimethylsulfoxide (DMSO) to make stock concentration of 10 mM and kept at −20°C. The 3-(4,5-dimethylthiahiazozy1)-3,5-di-phenytetrazoliumromide (MTT) was purchased from Sigma. Bortezomib was attained from Millennium Pharmaceuticals Inc. The Annexin V/propidium iodide (PI) Apoptosis Detection kit was obtained from BD Biosciences (San Jose, CA, USA).

**Table 1:** Cytotoxicity against NCI-H1975 cancer cell line and EGFR inhibitory activity of Gefitinib and its analogues.

Compounds	Structure	Molecular weight	IC_50_	
			NCI-H1975 cells (µM)	EGFR kinase (nM)
Gefitinib	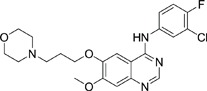	446.9	>10	11.2
V1801	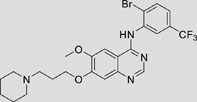	538.1	3.0	701.9
V1802	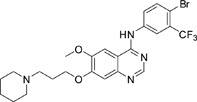	538.1	7.8	622.7
V1803	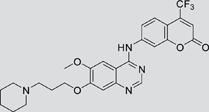	538.2	Insoluble	–
V1804	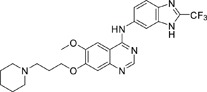	500.2	>10	2001.6
V1805	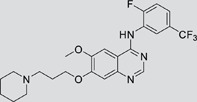	478.2	>10	119.6
V1806	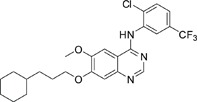	493.2	>10	>10000
V1807	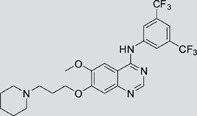	528.2	7.5	>10000
V1808	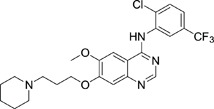	494.1	18.2	2665.8
V1301	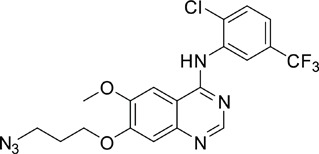	452.1	>10	>10000
V1302	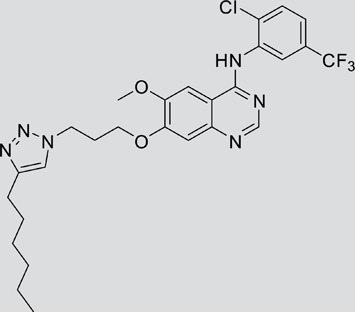	562.2	>10	>10000
V1303	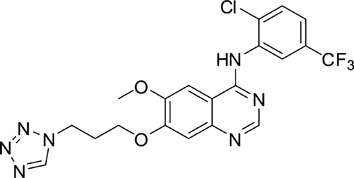	479.1	>10	>10000
V1304	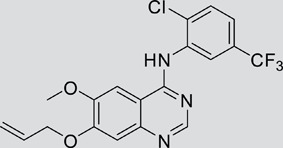	409.1	ND	>10000
V1305	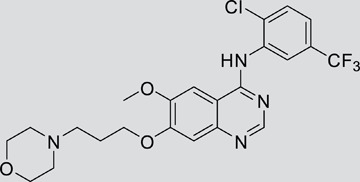	496.1	ND	>10000
V1306	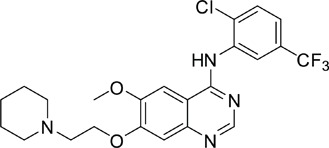	480.2	ND	>10000

ND: Not determined.

### Antibodies

The antibodies used in this study were as follows: anti-β-Actin (Sigma); anti-Casp-9 (C9), anti-Casp-8 (1C12), anti-Casp-3 (3G2), anti-PARP, anti-phospho-p44/42 MAPK, anti-EGFR (L858R), anti-STAT3, anti-phospho-STAT3 (Cell signaling); anti-c-Myc, anti-Noxa, anti-phospho-EGFR (Y1173), anti-pAKT and AKT (Santa Cruz Biotechnology); anti-ERK2 (Abcam); anti-rabbit or anti-mouse HRP-conjugated secondary antibody (Pierce). Detection was performed by using a Chemiluminescent Western detection kit (Cell Signaling).

### Cell culture

The lung cancer cell lines NCI-H1975, A549 and HCC827 were obtained from the American Tissue Culture Collection. A549 cells were cultured in Dulbecco modified Eagle medium (DMEM) containing 10% fetal bovine serum (FBS; Gibco/BRL, Grand Island, NY), 100 U/ml penicillin, 100 mg/ml streptomycin. NCI-H1975 and HCC827 cells were cultured in RPMI 1640 supplemented with 10% FBS, 100 U/ml penicillin and 100 mg/ml streptomycin.

### RNA preparation and RT–PCR

The total RNA of the cells was isolated using the TRIZOL Reagent (Invitrogen) and the phenol-chloroform extraction method according to the manufacturer's instruction. Total RNA (2 µg) was annealed with random primers at 65°C for 5 min. The cDNA was synthesized using a 1st-STRAND cDNA Synthesis Kit (Fermentas). For PCR amplification, primers are as follows: Actin, forward primer: 5′-CTCCTCCTGAGCGCAAGTACTC′-3′, reverse primer: 5′-TCCTGCTTGCTGATCCACATC′-3′; Puma, forward primer: 5′-GTCCTCAGCCCTCGCTCT-3′; reverse primer: 5′-CTAATTGGGCTCCATCTCG-3′; Bim, forward primer: 5′-GCCCCTACCTCCCTACAGAC-3′; reverse primer: 5′-ATGGTGGTGGCCATACAAAT-3′; Bcl-xl, forward primer: 5′-GGTGGGAGGGTAGAGTGGATGGT-3′; reverse primer: 5′-GGAAAGCGTAGACAAGGAGATGC-3′; Mcl-1, forward primer: 5′-CACGAGACGGTCTTCCAAGGCATGCT-3′; reverse primer: 5′-CTAGGTTGCTAGGGTGCAACTCTAGGA-3′; Noxa, forward primer: 5′-AAGAAGGCGCGCAAGAAC-3′; reverse primer: 5′-CGTGCACCTCCTGAGAAAAC-3′.

### Western blot assay

Cells were suspended in lysis buffer (50 mM Tris-HCl (pH 7.4), 150 mM NaCl, 1% triton X-100, 1% sodium deoxycholate, 0.1% SDS, 1 mM Na_3_VO_4_, 1 mM NaF, 1 mM EDTA, 1 mM PMSF, complete protease inhibitor cocktail) and cleared by centrifugation to obtain whole-cell lysates. Equal amounts of samples were separated by SDS-PAGE, transferred to nitrocellulose and immunoblotted with antibody indicated [27].

### Preparation of cytoplasmic fractions

For the detection of cytochrome c released from the mitochondria, cytoplasmic fractions were collected from NCI-H1975 cells after incubation with 0.1 mg/ml digitonin for 3 min at 37°C in Cytosol Lysis Buffer (0.01% digitonin, 1 mM EDTA, 1 mM EGTA, 1 mM PMSF, protease inhibitors cocktails in 1×PBS at pH 7.4). After centrifugation, aliquots of the supernatant (cytoplasmic fraction) and the pellet containing the mitochondria were analyzed by Western blotting.

### Assessment of cell proliferation and apoptosis

Cells were treated with V1801 or gefitinib for indicated concentrations and time points. Cell proliferation was determined using MTT assay. Cell viability was estimated by trypan blue dye exclusion [27]. Externalization of phosphatidylserine was tested using an Annexin V/PI Apoptosis Detection kit (BD Biosciences, San Jose, CA) according to manufacturer's instruction. After staining with Hoechst 33258 at 10 µg/ml for 10 min, cell death was assessed by a fluorescence microscope (Leica).

### In vitro EGFR kinase assays

In vitro kinase inhibitory ability was determined using the ADP-Glo™ Kinase Assay Kit and EGFR Kinase Enzyme System (Promega), following the manufacturer's instructions.

### siRNA assays

Using HiPerFect Transfection Reagent (Qiagen, Crawley, UK) according to the manufacturer's protocol, cells were transfected with 100 nM double-stranded siRNA oligonucleotides. The siRNA sequences were 5′-GGUGCACGUUUCAUCAAUU-3′ (Noxa siRNA1), 5′-AACUUCCGGCAGAAACUUC-3′ (Noxa siRNA2), and 5′-UUCUCCGAACGUGUCACGUTT-3′ (negative control (NC) siRNA), 5′-CAGAAATGTCCTGAGCAAT-3′ (c-Myc siRNA1), 5′-AAGGTCAGAGTCTGGATCACC-3′ (c-Myc siRNA2).

### Clonogenic assay

After incubation with V1801 (3 µM) or gefitinib (3 µM) for 12 h, a soft-agar colony assay was performed. To this end, trypsinized cells were suspended in RPMI 1640 medium containing 10% FBS and low-melting-point 0.3% agarose (Amresco, Solon, OH) and subsequently overlaid onto a solidified layer of RPMI 1640 medium containing 10% FBS and 0.6% agarose in 35 mm plate (5,000 cells/plate) in triplicate. After 2 weeks, the colonies were fixed in 90% ethanol and stained with 0.005% crystal violet (Sigma). The colony forming units with more than 100 cells were counted using a light microscope [28].

### Combination index

The interaction between compounds was quantified by determining the combination index (CI) calculated by the Chou–Talalay equation [29], [30]. The general equation for the classic isobologram is given by: CI = (D)1/(Dx)1+(D)2/(Dx)2. Where Dx indicates the dose of one compound alone required to produce an effect, (D)1 and (D)2 are the doses of compounds 1 and 2, respectively, necessary to produce the same effect in combination. From this analysis, the combined effects of the two compounds can be summarized as follows: CI<1 indicates synergism; CI = 1 indicates additive effect; and CI>1 indicates antagonism.

### Murine models

All animal studies were conducted according to protocols approved by the Animal Ethics Committee of the Institute of Zoology, Chinese Academy of Sciences, with the approval ID of AEC2011030205. All mice used in this study were bred and maintained in a specific pathogen-free environment. NCI-H1975 cells (2.5×10^6^) were injected subcutaneously into the right flank of nude mice. When tumor reached a palpable size, animals were randomized into 3 groups (n = 11 for each group) and treated with V1801 (30 mg/kg/day), gefitinib (30 mg/kg/day) or vehicle control for 3 weeks. Animals were sacrificed when tumors reached 2 cm or if the mice appeared moribund to prevent unnecessary morbidity to the mice. At the time of the animals' death, tumors were excised; cells were separated and lyzed for Western blotting using anti-Noxa antibody and anti-Actin antibodies. Noxa expression was then quantified by densitometry analysis and normalized against Actin expression.

### Statistical analysis

All experiments were repeated at least three times and the data were presented as the mean±SD unless noted otherwise. *P* values less than 0.05 indicate statistical significance.

## Results

### Effects of gefitinib mimetics on lung cancer cells and EGFR kinase activity

In this work, fourteen gefitinib analogues were synthesized by exchanging the positions of the C5 and C6 substituents and varying the C4-amino functionality of the quinazoline core of gefitinib, and their effects on NCI-H1975 cells were evaluated using gefitinib as a control (Table 1). Among these compounds, V1801, V1802 and V1807 were found to exhibit much more potent cytotoxicity against NCI-H1975 cells than gefitinib, and V1801 is the most potent one, whose IC50 value of 3 µM is more than three folds lower than that of gefitinib (Table 1). Generally, introduction of a trifluoromethylaniline moiety to the C4 position of the quinazoline core is an effective manner of improving the cytotoxicity. A bromine substituent at the C2' position was found to be a suitable modification for higher activity (V1801 vs. V1802, V1805 and V1808). In addition, the piperidine moiety at the terminal of C6-substituent of these compounds is also superior by comparison with the corresponding morpholine, cyclohexane, azido, triazole and tetrazole functionalities.

**Figure 1:**
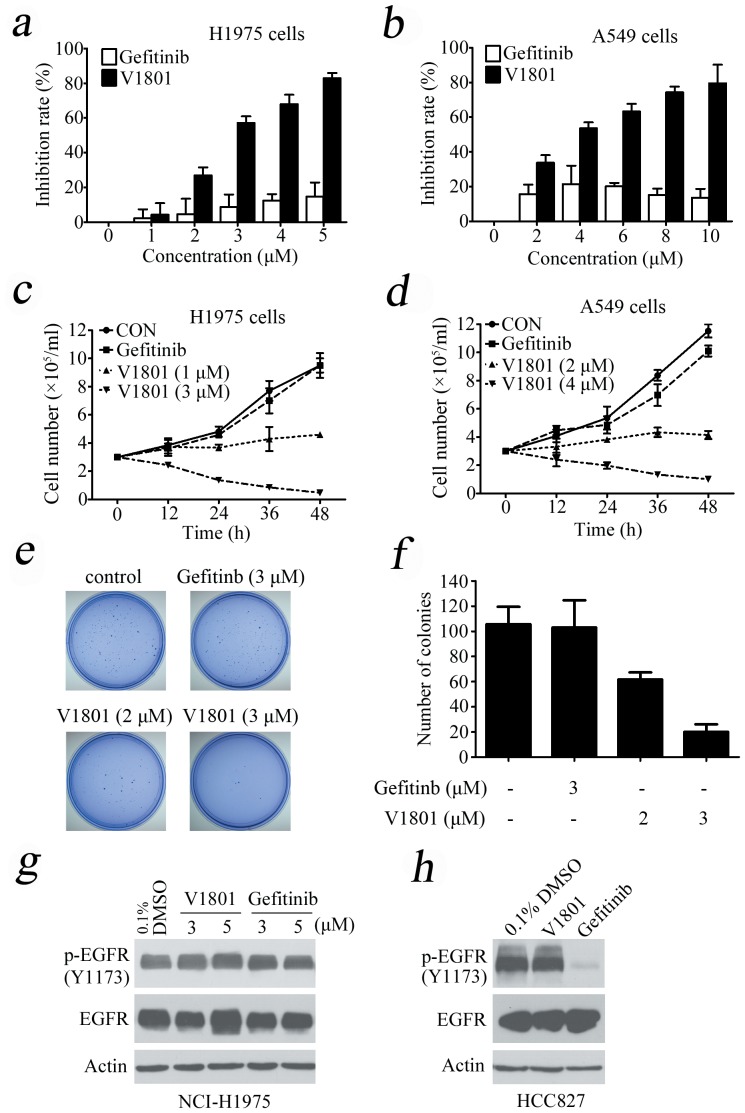
Effects of V1801 on NSCLC cells. (a, b) The cells were treated with gefitinib or V1801 for 48 h, and analyzed by MTT assay. (c, d) The cells were treated with gefitinib or V1801 for indicated time points and assessed by trypan blue exclusion analysis. (e) Soft-agar colony formation assay for NCI-H1975 cells treated with or without V1801 or gefitinib. (f) Quantitation of foci counting. The mean+SD of three independent experiments is shown. (g, h) NCI-H1975 (g) and HCC827 (h) cells were treated with V1801 or gefitinib for 12 h, lysed, and Western blot assay was performed using indicated antibodies.

Using 3-(4,5-dimethylthiazol-2-yl)-2,5-diphenyltetrazolium bromide (MTT) assay, we showed that V1801 displayed significant inhibitory effects against the proliferation of NCI-H1975 (Fig. 1a) and wild type EGFR-expressing A549 (Fig. 1b) cells in a dose-dependent fashion. By trypan blue exclusion analysis, we found that V1801 rapidly reduced viable NCI-H1975 (Fig. 1c) and A549 (Fig. 1d) cells in a dose- and time-dependent manner. We assessed V1801's effects on colony formation activity of the cells, and reported that as compared to gefitinib or vehicle control, V1801 drastically inhibited the clonogenic activity of NCI-H1975 cells (Figs. 1e and 1f).

We tested the effects of these gefitinib analogues on EGFR and showed that V1801, V1802 and V1805 exerted moderate inhibitory activity on EGFR kinase activity (Table 1). V1801 had an IC_50_ on EGFR much higher than that of gefitinib (701.9 nM vs 11.2 nM; Table 1), indicating that the new modifications made in analogue V1801 are workable but less superior for inhibiting EGFR kinase activity. By Western blot analysis in NCI-H1975 cells, both gefitinib and V1801 failed to down-regulate phosphorylated EGFR (p-EGFR) (Fig. 1g), while in gefitinib-sensitive HCC827 cells, gefitinib but not V1801 induced de-phosphorylation of p-EGFR (Fig. 1h). These results indicate V1801 might induce cell apoptosis via a mechanism independent of EGFR signaling pathway inhibition.

### V1801 induces apoptosis of NSCLC cells in a caspases-dependent manner

**Figure 2:**
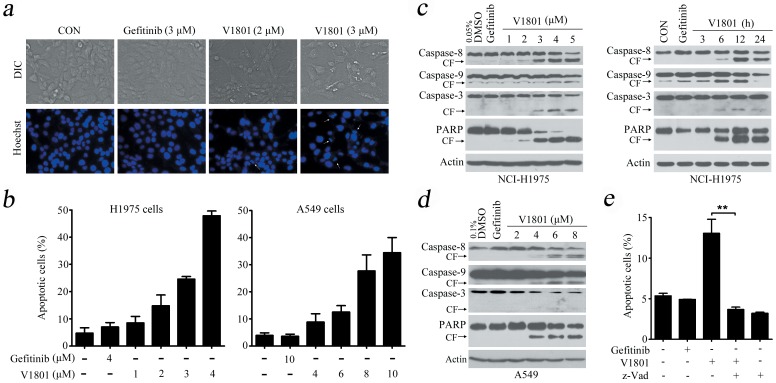
V1801 induces apoptosis of NSCLC cells. (a) NCI-H1975 cells were treated with gefitinib or V1801 for 24 h, and evaluated by Heochst33258 assay. (b) The cells were treated with gefitinib or V1801 for 24 h, and apoptotic cell death was analyzed by Annexin V/PI staining and flow cytometry. (c) NCI-H1975 cells were treated with V1801 or gefitinib (3 µM), lysed, and Western blot assay was performed using indicated antibodies. (d) A549 cells were treated with V1801 or gefitinib (3 µM) for 24 h, lysed, and Western blot assay was performed using indicated antibodies. (e) NCI-H1975 cells were pre-treated with z-VAD-fmk (20 µM) for 1 h followed by treatment with V1801 at 3 µM for 12 h, and the apoptotic cells were determined by Annexin V/PI staining and flow cytometry analysis. The mean+SD of three independent experiments is shown. **, *P*<0.01.

We next tested whether or not V1801 induces apoptosis of the cells. By Hoechst 33258 staining, it was showed in NCI-H1975 cells V1801 caused chromatin condensation and fragmentation which are typical apoptotic nuclear morphological changes (Fig. 2a). An annexin V/propidium iodide (PI)-staining and flow cytometry assays confirmed that treatment with V1801 for 24 h induced apoptosis in NCI-H1975 and A549 cells (Fig. 2b). By Western blot analysis, V1801 treatment was found to result in a marked increase in the active form of caspase-9, caspase-8 and caspase-3 and cleavage of poly(ADP-ribose) polymerase (PARP) in NCI-H1975 cells in a dose- and time-dependent fashion (Fig. 2c) and in A549 cells (Fig. 2d). When NCI-H1975 cells were pre-treated with a pan-caspase inhibitor benzyloxycarbonyl-Val-Ala-Asp fluoromethylketone (z-VAD.fmk; 20 µM) for 1 h followed by treatment with V1801 at 3 µM for 12 h, V1801-induced apoptosis was suppressed (Fig. 2e), indicating that the activation of caspase cascade is essential for V1801-induced apoptosis in gefitinib-resistance cells.

### V1801 up-regulates Noxa in NSCLC cells

**Figure 3:**
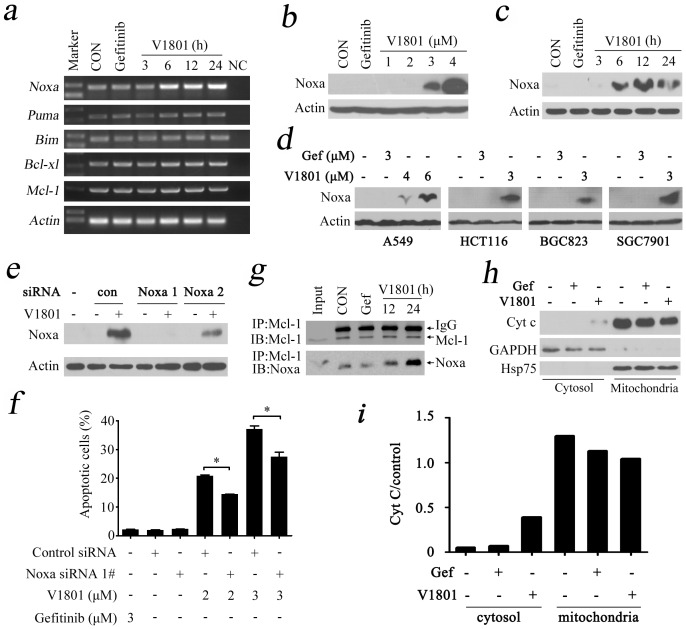
Noxa plays a critical role in V1801-triggered apoptosis of gefitinib-resistant NSCLC cells. (a) RT-PCR analysis of the expression of *Noxa*, *Puma*, *Bim*, *Bcl-xl*, and *Mcl-1* at mRNA level in NCI-H1975 cells treated with gefitinib (3 µM) or V1801 (3 µM) for indicated time points. (b) NCI-H1975 cells were treated with V1801 at indicated concentration for 24 h, lysed, and analyzed by Western blot analysis using an anti-Noxa antibody. (c) NCI-H1975 cells were treated with V1801 at 3 µM for indicated time points, lysed, and analyzed by Western blot analysis using an anti-Noxa antibody. (d) Indicated cells were treated with V1801 or gefitinib for 24 h, lysed, and analyzed by Western blot analysis. (e) NCI-H1975 cells transfected with control or Noxa specific siRNA were treated with or without V1801 (3 µM) for 24 h, lysed, and Western blot analysis was performed. (f) Cells were transfected with siRNA and treated with V1801 as described in (e), then analyzed by Annexin V/PI staining and flow cytometry. (g) NCI-H1975 cells were treated with gefitinib or V1801, lysed, and immunoprecipitation/Western blot assays were performed using indicated antibodies. (h, i) NCI-H1975 cells were treated with V1801 or gefitinib for 24 h, lysed, cytosol and mitochondrial fractions were isolated by centrifugation, and Western blotting was conducted using indicated antibodies (h). The expression of cytochrome C was quantified by densitometry analysis and normalized against GAPDH or Hsp75 (i).

To elucidate the mechanism of action of V1801, its effects on the expression of antiapoptotic proteins (Bcl-xl and Mcl-1) and proapoptotic proteins (Noxa, Puma and Bim) of Bcl-2 family were tested in NSCLC cells with RT-PCR analysis. Interestingly, we found that V1801 strongly increased the expression of Noxa in a time-dependent manner (Fig. 3a). We then evaluated the effect of V1801 on protein level of Noxa, and found that treatment with V1801 at 3 to 4 µM for 24 h markedly up-regulated Noxa in NCI-H1975 cells (Fig. 3b). In NCI-H1975 cells treated with V1801 at 3 µM for 3 to 6 h, Noxa was drastically increased (Fig. 3c). In A549, HCT116, BGC823 and SGC7901 cells, treatment with V1801 at 3 to 6 µM for 24 h also up-regulated Noxa (Fig. 3d). However, gefitinib failed to up-regulate Noxa in these cell lines (Figs. 3a through 3d), indicating that these two compounds have totally distinct mechanisms in inducing apoptosis of lung cancer cells.

### Up-regulation of Noxa is required for V1801-induced apoptosis of NSCLC cells

To evaluate its role in V1801-induced apoptosis, Noxa was silenced by specific siRNA (Fig. 3e), and the cells upon V1801 were analyzed by Annexin V/PI staining and flow cytometry. We found that knockdown of Noxa significantly attenuated V1801-induced apoptosis of NCI-H1975 cells (Fig. 3f), indicating that up-regulation of Noxa is essential for V1801-induced apoptosis. However, Noxa silencing could not completely abrogated V1801-induced cell apoptosis (Fig. 3f), suggesting that other factors might be involved in programmed cell death triggered by this gefitinib analogue.

Noxa can directly bind Mcl-1 and competitively release Bim or Bak from this apoptosis antagonist, leading to mitochondrial dysfunction and initiation of programmed cell death [18], [31]. We performed co-immunoprecipitation and Western blot assays in V1801-treated NCI-H1975 cells, and found that V1801 but not gefitinib enhanced Mcl-1-Noxa binding affinity (Fig. 3g). We then examined the subcellular localization of cytochrome c by Western blot, while the expression of cytochrome C was quantified by densitometry analysis and normalized against GAPDH or Hsp75. We found that in NCI-H1975 cells treated with V1801 (3 µM) but not gefitinib (3 µM) for 24 h, cytochrome c was partially translocated from mitochondria to cytosol (Fig. 3h and i). These results illustrate that increased expression of Noxa mediates apoptosis induced by V1801 through mitochondria pathway.

### Investigating the mechanisms underlying V1801-induced Noxa up-regulation

**Figure 4:**
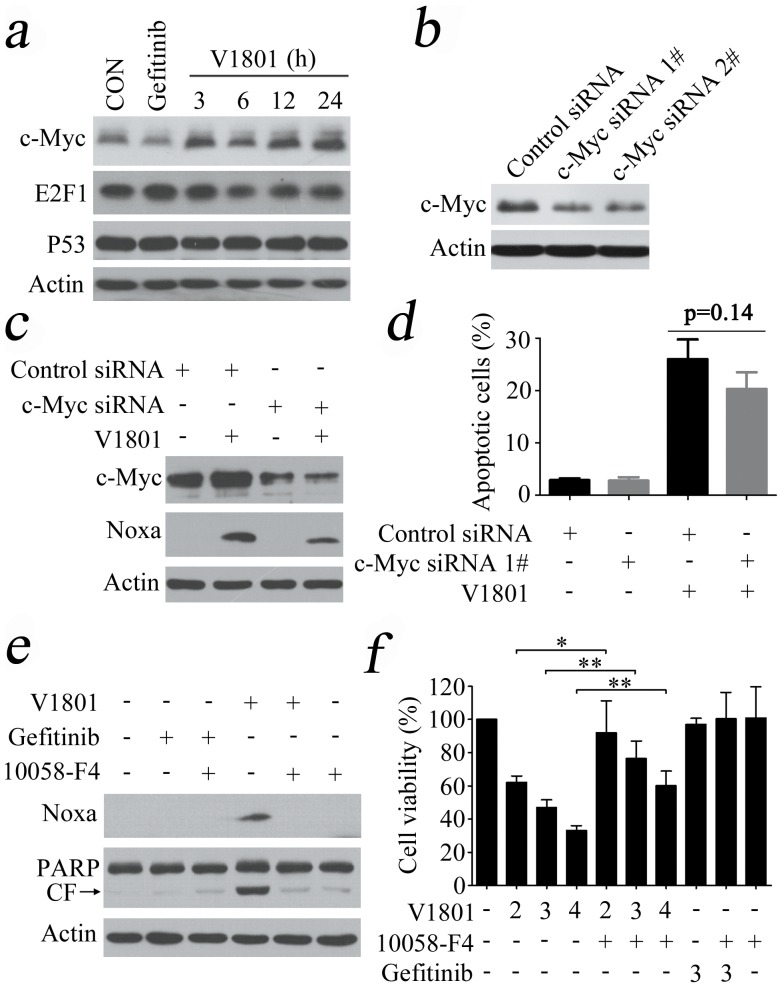
c-Myc's role in V1801-induced Noxa up-regulation. (a) NCI-H1975 cells were treated with gefitinib (3 µM) or V1801 (3 µM) for indicated time points, lysed, and Western blot assay was conducted using indicated antibodies. (b) Western blot analysis using lysates of NCI-H1975 cells transfected with control or c-Myc specific siRNA and anti-c-Myc antibody. (c) NCI-H1975 cells transfected with c-Myc specific siRNA were treated with or without V1801, lysed, and Western blotting was conducted using indicated antibodies. (d) NCI-H1975 cells transfected with c-Myc specific siRNA were treated with or without V1801, and evaluated by Anexin V/PI staining and flow cytometry. (e) NCI-H1975 cells were treated with indicated protocols for 24 h, lysed, and Western blot analysis was performed. (f) NCI-H1975 cells were treated with indicated protocols for 24 h, and assessed by MTT assay. *, *p*<0.05; **, *p*<0.01.

Studies have shown that the p53, c-Myc and E2F1 can transcriptionally increase Noxa expression via direct binding to Noxa promoter [32]. We tested the expression of these transcription factors, and reported that only c-Myc was upregulated in NCI-H1975 cells treated with V1801 at 3 µM for 3 h (Fig. 4a). To determine its role in V1801-induced Noxa up-regulation and NCI-H1975 cell apoptosis, c-Myc was silenced by specific siRNA (Fig. 4b). We found that c-Myc silencing slightly suppressed V1801-caused Noxa expression (Fig. 4c) and programmed cell death in NCI-H1975 cells (Fig. 4d). To confirm these results, the c-Myc inhibitor 5-(4-ethyl-benzylidene)-2-thioxothiazolidin-4-one (10058-F4) [33] was employed to inhibit c-Myc transcription function. The results showed that 10058-F4 partially repressed V1801-induced Noxa up-regulation (Fig. 4e) and cytotoxicity in NCI-H1975 cells (Fig. 4f).

**Figure 5:**
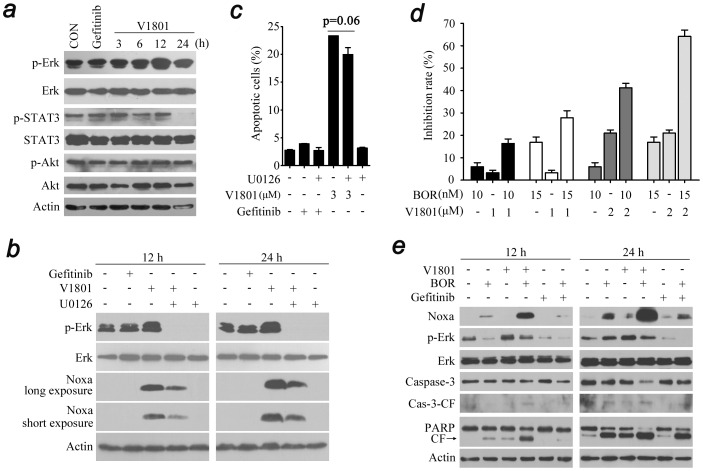
V1801 interferes with Erk signal in NCI-H1975 cells. (a) NCI-H1975 cells were treated with gefitinib (3 µM) or V1801 (3 µM) for indicated time points, lysed, and Western blot analysis was performed. (b) NCI-H1975 cells were treated with indicated protocols for 12 or 24 h, lysed, and Western blot analysis was performed. (c) NCI-H1975 cells were treated with indicated protocols for 24 h, and apoptotic cell death was evaluated by Annexin V/PI staining and flow cytometry. (d) NCI-H1975 cells were treated for 24 h with BOR and/or V1801, and then assessed by MTT assay. (e) NCI-H1975 cells were treated with V1801 (2 µM), gefitinib (2 µM), BOR (10 nM), or their combinations for indicated time points. The cells were then lysed and subjected to Western blotting using indicated antibodies.

Previous studies showed that cisplatin can up-regulate Noxa in Erk dependent fashion, and inhibition of Erk by siRNA or small compound inhibitor U0126 markedly attenuated cisplatin-induced cell death as well as Noxa expression [15]. We tested the effects of V1801 on Erk, and found that in NCI-H1975 cells treated with V1801 at 3 µM for 12 h, the phospho-Erk (p-Erk) was increased (Fig. 5a). Treated with V1801 for 24 h also down-regulated the phospho-signal transducers and activators of transcription 3 (p-STAT3) but not phospho-Rac-serine/threonine-protein kinase (p-Akt) (Fig. 5a). It was showed that U0126 could attenuate V1801-induced Noxa expression (Fig. 5b) and slightly (p = 0.06) reduced V1801-induced apoptosis of NCI-H1975 cells (Fig. 5c).

In mantle-cell lymphoma, Noxa activation is required for the proteasome inhibitor bortezomib induced apoptosis [13]. We tested the combined effect of V1801 and bortezomib on NCI-H1975 cells and found that bortezomib at 10 to 15 nM significantly enhanced V1801 (2 µM)-caused inhibition of cell proliferation (Fig. 5d). An analysis of the combination index (CI) [29], [30] indicated that the CI values were less than 1, indicating that these two agents could exert synergistic effects on NSCLC cells. At molecular level, we showed that treatment of NCI-H1975 cells with V1801 and bortezomib resulted in enhanced up-regulation of Noxa and activation of Casp-3 and cleavage of PARP (Fig. 5e). However, the up-regulation of p-Erk induced by V1801 was slightly attenuated by bortezomib (Fig. 5e).

### *In vivo* anti-tumor efficacy of V1801 on xenograft mouse model

**Figure 6:**
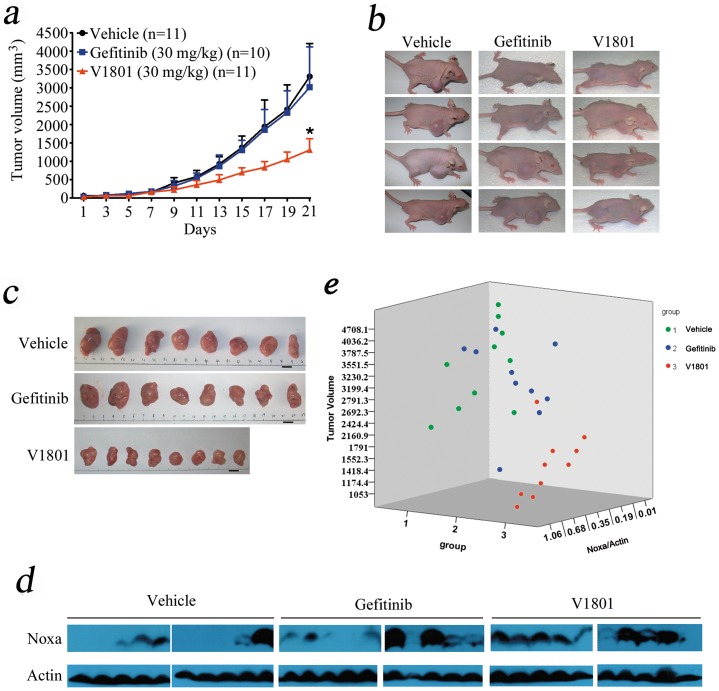
*In vivo* therapeutic efficacy of V1801 on mice bearing gefitinib-resistant NSCLC cells. (a) Nude mice inoculated subcutaneously with NCI-H1975 cells were treated with gefitinib or V1801 for 3 weeks, and caliper measurements of the longest perpendicular tumor diameters were performed every two days to estimate the tumor volume. (b) Images of mice two weeks after initiation of the treatment. (c) Images of xenograft tumors obtained from mice. Eight representative tumors for each treatment group are shown. (d, e) Western blot analysis of lysates of tumor samples using indicated antibodies (d). Noxa expression was quantified by densitometry analysis and normalized against Actin expression (e).

We tested the *in vivo* anti-tumor efficacy of V1801. To do this, nude mice were subcutaneously inoculated into the right flank with 2.5×10^6^ NCI-H1975 cells in 0.1 ml PBS. When the tumors reached a palpable size, the mice were randomized into 3 groups (n = 11 for each group) and oral administration with V1801 (30 mg/kg/day), gefitinib (30 mg/kg/day) or vehicle control for 3 weeks. Intriguingly, we found that V1801 significantly inhibited tumor growth compared to vehicle control or gefitinib (P<0.05) (Figs. 6a through 6c). Animals were euthanized when they were treated for 3 weeks and the tumor samples were isolated and imaged (Fig. 6c). Proteins were extracted from tumor samples and Western blot assay was conducted. We found that in samples from mice treated with V1801, the expression of Noxa was up-regulated as compared to that in samples separated from vehicle or gefitinib control mice (Fig. 6d and e). These results demonstrate that administration of V1801 exhibits tremendous therapeutic potentials.

## Discussion

EGFR is a cell-surface receptor activated in more than half of patients with NSCLC, and this activation can be the result of protein over-expression, increased gene copy number, or genetic mutations [2]. Gefitinib's anti-lung cancer effects attribute to its binding to the adenosine triphosphate binding site in the tyrosine kinase domain of EGFR, thereby inhibiting EGFR kinase activity [3], [5], [34], [35]. Although many patients initially respond to gefitinib, resistance and tumor recurrence eventually develop. To overcome such drug resistance, novel mutant-selective EGFR kinase inhibitors against EGFR T790M were reported [36], and 4-pyrrylamino quinazolines as new gefitinib analogues were synthesized [37]. In this study, we design, synthesize and evaluated a number of novel quinazoline derivatives by exchanging the positions of the C5 and C6 substituents and varying the C4-amino functionality of gefitinib, and report that compound V1801 having a trifluoromethyl group at the C5'-position and a bromine at the C2'-position of the aniline moiety substituted at the C4 position of quinazoline core overcomes gefitinib resistance via up-regulation of Noxa, suggesting a novel strategy to fight against NSCLC with EGFR T790M mutation.

The mitochondrial pathway of apoptosis constitutes one of the main safeguards against tumorigenesis, and the Bcl-2 family includes the central players of this pathway that regulate cell fate through the control of mitochondrial outer membrane permeabilization. Therefore, Bcl-2 family antagonists have been developed for cancer therapy [38]. To inhibit Mcl-1 pro-survival function, occlusion of Mcl-1 hydrophobic groove by BH3-only proteins represents an effective strategy [39]. Noxa belongs to the BH3-only proteins of the Bcl-2 family and is able to interact with Mcl-1, leading to initiation of apoptosis signals [31], [40], [41]. Compared with the parental compound gefitinib, V1801 is a relatively weak EGFR inhibitor which bears a much more potent cytotoxicity in T790M EGFR-harboring NCI-H1975 cells (Table 1). These results suggest that V1801 may have “off-target” effects in gefitinib-resistance NSCLC cells. We carry out experiments to investigate the mechanisms of action of V1801 by measuring the expression of Bcl-2 family members including Noxa, Puma, Bim, Bcl-xl and Mcl-1, and found that in NCI-H1975 and A549 cells upon V1801, Noxa is up-regulated at an early stage (3 h) at both mRNA and protein levels (Figs. 3a through 3d). In colon, gastric cancer cells and melanoma cells, V1801 also induces up-regulation of Noxa (Fig. 3d), suggesting that this mechanism is valid in a spectrum of cancer cells. While the expression of Mcl-1 in NCI-H1975 cells is not affected by V1801 treatment, Mcl-1-Noxa interaction is enhanced (Figs. 3a and 3g). Consequently, cytochrome C partially translocates from mitochondria to cytosol (Fig. 3h and i). These results indicate that V1801 can induce Noxa expression and trigger intrinsic apoptotic pathway, leading to activation of effector caspases and cleavage of PARP (Fig. 2). Interestingly, V1801 can also activate caspase-8 (Fig. 2), demonstrating that V1801 also activates the extrinsic apoptotic pathway and the mechanisms of action of this compound in inducing apoptosis are complicated and warrant further investigation.

The expression of Noxa is regulated by transcription factors HIF-1α, E2F-1 and p53 [32]. Recent studies show that in a variety of tumor cell types, the induction of Noxa by proteasome inhibitor bortezomib is directly dependent on the oncogene c-Myc [22]. Indeed, c-Myc not only regulates those genes capable of promoting cell growth and metabolism, but also activates apoptosis initiators [42]. c-Myc amplifies mitochondrial apoptotic pathway by inhibiting anti-apoptotic Bcl-2 proteins and activating pro-apoptotic Bcl-2 family members such as BH3-only proteins [43]. Here we report that c-Myc protein is up-regulated by V1801 treatment in NSCLC cells (Fig. 4a). However, c-Myc silencing by siRNA only slightly but not significantly (p = 0.14) attenuates V1801-caused programmed cell death of NSCLC cells (Fig. 4d). Given that Erk pathway can mediate Noxa up-regulation [15] and Erk itself has pro-apoptotic function [15], [44], [45], we test the effect of V1801 on Erk expression and report that this gefitinib analog increases p-Erk, but Erk inhibition only slightly (p = 0.06) suppresses V1801-induced apoptosis of H1975 cells (Fig. 5c). The results also suggest the complexity of the mechanisms of action of this compound in inducing apoptosis of lung cancer cells. In myeloma cells, bortezomib inactivates p-Erk and synergistically potentiates the apoptotic effects of a novel compound 6-O-Angeloylplenolin [46]. Interestingly, we show that while bortezomib significantly enhances V1801-induced cytotoxicity (Fig. 5d) and up-regulation of Noxa (Fig. 5e) on NCI-H1975 cells, it antagonizes V1801-caused p-Erk activation (Fig. 5e). These results indicate that bortezomib, as a multi-target agent, may increase Noxa expression through other unidentified mechanism which warrants further investigation, and combined use of V1801 and bortezomib may have therapeutic potential in gefinitib-resistance NSCLC cells.

The *in vivo* studies show that in nude mice inoculated with NCI-H1975 cells, treatment with V1801 at 30 mg/kg per day significantly inhibits tumor growth, while gefitinib at the same dosage does not show therapeutic benefit (Fig. 6a through 6c). V1801 also up-regulates Noxa *in vivo* (Fig. 6d), and V1801-treated mice have smaller tumors and higher Noxa expression compared to those treated with vehicle control or Gefitinib (Fig. 6e). Therefore, our data indicates that gefinitib analogs with weak EGFR inhibitory activity can overcome TKI-resistance via activation of BH-3 only pro-apoptotic proteins, and V1801 represents a potential new therapeutic agent for patients with NSCLC.

## References

[1] Jemal A, Bray F, Center MM, Ferlay J, Ward E, et al (2011) Global cancer statistics. CA Cancer J Clin 61: 69–90. 21296855 10.3322/caac.20107

[2] Herbst RS, Heymach JV, Lippman SM (2008) Lung Cancer. N Engl J Med 359: 1367–1380. 18815398 10.1056/NEJMra0802714 PMC10662965

[3] Lynch TJ, Bell DW, Sordella R, Gurubhagavatula S, Okimoto RA, et al (2004) Activating Mutations in the Epidermal Growth Factor Receptor Underlying Responsiveness of Non-Small-Cell Lung Cancer to Gefitinib. N Engl J Med 350: 2129–2139. 15118073 10.1056/NEJMoa040938

[4] Ciardiello F, Tortora G (2008) EGFR Antagonists in Cancer Treatment. N Engl J Med 358: 1160–1174. 18337605 10.1056/NEJMra0707704

[5] Shepherd FA, Rodrigues Pereira J, Ciuleanu T, Tan EH, Hirsh V, et al (2005) Erlotinib in Previously Treated Non-Small-Cell Lung Cancer. N Engl J Med 353: 123–132. 16014882 10.1056/NEJMoa050753

[6] Pao W, Miller VA, Politi KA, Riely GJ, Somwar R, et al (2005) Acquired Resistance of Lung Adenocarcinomas to Gefitinib or Erlotinib Is Associated with a Second Mutation in the EGFR Kinase Domain. PLoS Med 2: e73 doi:10.1371/journal.pmed.0020073 15737014 10.1371/journal.pmed.0020073 PMC549606

[7] Kobayashi S, Boggon TJ, Dayaram T, Janne PA, Kocher O, et al (2005) EGFR mutation and resistance of non-small-cell lung cancer to gefitinib. N Engl J Med 352: 786–792. 15728811 10.1056/NEJMoa044238

[8] Yun CH, Mengwasser KE, Toms AV, Woo MS, Greulich H, et al (2008) The T790M mutation in EGFR kinase causes drug resistance by increasing the affinity for ATP. Proc Natl Acad Sci U S A 105: 2070–2075. 18227510 10.1073/pnas.0709662105 PMC2538882

[9] Kim Y, Ko J, Cui Z, Abolhoda A, Ou S-HI, et al (2012) The EGFR T790M mutation in acquired resistance to an irreversible second-generation EGFR inhibitor. Mol Cancer Ther 10.1158/1535-7163.MCT-11-0750. 10.1158/1535-7163.MCT-11-0750 22228822

[10] Hanahan D, Weinberg RA (2011) Hallmarks of Cancer: The Next Generation. Cell 144: 646–674. 21376230 10.1016/j.cell.2011.02.013

[11] Chipuk JE, Moldoveanu T, Llambi F, Parsons MJ, Green DR (2010) The BCL-2 family reunion. Mol Cell 37: 299–310. 20159550 10.1016/j.molcel.2010.01.025 PMC3222298

[12] Youle RJ, Strasser A (2008) The BCL-2 protein family: opposing activities that mediate cell death. Nat Rev Mol Cell Biol 9: 47–59 10.1038/nrm2308. 18097445 10.1038/nrm2308

[13] Perez-Galan P, Roue G, Villamor N, Montserrat E, Campo E, et al (2006) The proteasome inhibitor bortezomib induces apoptosis in mantle-cell lymphoma through generation of ROS and Noxa activation independent of p53 status. Blood 107: 257–264. 16166592 10.1182/blood-2005-05-2091

[14] Kim JY, Ahn HJ, Ryu JH, Suk K, Park JH (2004) BH3-only protein Noxa Is a mediator of hypoxic cell death induced by hypoxia-inducible factor 1α. J Exp Med 199: 113–124. 14699081 10.1084/jem.20030613 PMC1887730

[15] Sheridan C, Brumatti G, Elgendy M, Brunet M, Martin SJ (2010) An ERK-dependent pathway to Noxa expression regulates apoptosis by platinum-based chemotherapeutic drugs. Oncogene 29: 6428–6441. 20802529 10.1038/onc.2010.380

[16] Oda E, Ohki R, Murasawa H, Nemoto J, Shibue T, et al (2000) Noxa, a BH3-Only Member of the Bcl-2 Family and Candidate Mediator of p53-Induced Apoptosis. Science 288: 1053–1058. 10807576 10.1126/science.288.5468.1053

[17] Schuler M, Maurer U, Goldstein JC, Breitenbucher F, Hoffarth S, et al (2003) p53 triggers apoptosis in oncogene-expressing fibroblasts by the induction of Noxa and mitochondrial Bax translocation. Cell Death Differ 10: 451–460. 12719722 10.1038/sj.cdd.4401180

[18] Willis SN, Chen L, Dewson G, Wei A, Naik E, et al (2005) Proapoptotic Bak is sequestered by Mcl-1 and Bcl-xL, but not Bcl-2, until displaced by BH3-only proteins. Genes Dev 19: 1294–1305. 15901672 10.1101/gad.1304105 PMC1142553

[19] Lowman XH, McDonnell MA, Kosloske A, Odumade OA, Jenness C, et al (2010) The Proapoptotic Function of Noxa in Human Leukemia Cells Is Regulated by the Kinase Cdk5 and by Glucose. Mol Cell 40: 823–833. 21145489 10.1016/j.molcel.2010.11.035

[20] Elgendy M, Sheridan C, Brumatti G, Martin SJ (2011) Oncogenic Ras-Induced Expression of Noxa and Beclin-1 Promotes Autophagic Cell Death and Limits Clonogenic Survival. Mol cell 42: 23–35. 21353614 10.1016/j.molcel.2011.02.009

[21] Hershko T, Ginsberg D (2004) Up-regulation of Bcl-2 Homology 3 (BH3)-only Proteins by E2F1 Mediates Apoptosis. J Biol Chem 279: 8627–8634. 14684737 10.1074/jbc.M312866200

[22] Nikiforov MA, Riblett M, Tang WH, Gratchouck V, Zhuang D, et al (2007) Tumor cell-selective regulation of NOXA by c-MYC in response to proteasome inhibition. Proc Natl Acad Sci U S A 104: 19488–19493. 18042711 10.1073/pnas.0708380104 PMC2148316

[23] Wang Q, Mora-Jensen H, Weniger MA, Perez-Galan P, Wolford C, et al (2009) ERAD inhibitors integrate ER stress with an epigenetic mechanism to activate BH3-only protein NOXA in cancer cells. Proc Natl Acad Sci U S A 106: 2200–2205. 19164757 10.1073/pnas.0807611106 PMC2629785

[24] Phelps RM, Johnson BE, Ihde DC, Gazdar AF, Carbone DP, et al (1996) NCI-navy medical oncology branch cell line data base. J Cell Biochem 63 S24: 32–91 10.1002/jcb.240630505. 10.1002/jcb.240630505 8806092

[25] Sordella R, Bell DW, Haber DA, Settleman J (2004) Gefitinib-Sensitizing EGFR Mutations in Lung Cancer Activate Anti-Apoptotic Pathways. Science 305: 1163–1167. 15284455 10.1126/science.1101637

[26] Tang Z, Du R, Jiang S, Wu C, Barkauskas DS, et al (2008) Dual MET-EGFR combinatorial inhibition against T790M-EGFR-mediated erlotinib-resistant lung cancer. Br J Cancer 99: 911–922. 19238632 10.1038/sj.bjc.6604559 PMC2538758

[27] Fang HT, Zhang B, Pan XF, Gao L, Zhen T, et al (2012) Bortezomib interferes with C-KIT processing and transforms the t(8;21)-generated fusion proteins into tumor-suppressing fragments in leukemia cells. Proc Natl Acad Sci U S A 109: 2521–2526. 22308476 10.1073/pnas.1121341109 PMC3289299

[28] Ma L, Wen ZS, Liu Z, Hu Z, Ma J, et al (2011) Overexpression and Small Molecule-Triggered Downregulation of CIP2A in Lung Cancer. PLoS ONE 6: e20159. 21655278 10.1371/journal.pone.0020159 PMC3105001

[29] Hu Z, Pan XF, Wu FQ, Ma LY, Liu DP, et al (2009) Synergy between Proteasome Inhibitors and Imatinib Mesylate in Chronic Myeloid Leukemia. PLoS ONE 4: e6257. 19606213 10.1371/journal.pone.0006257 PMC2705802

[30] Chou TC, Talalay P (1981) Generalized equations for the analysis of inhibitions of Michaelis-Menten and higher-order kinetic systems with two or more mutually exclusive and nonexclusive inhibitors. Eur J Biochem 115: 207–216. 7227366 10.1111/j.1432-1033.1981.tb06218.x

[31] Han J, Goldstein LA, Hou W, Rabinowich H (2007) Functional Linkage between NOXA and Bim in Mitochondrial Apoptotic Events. J Biol Chem 282: 16223–16231. 17374615 10.1074/jbc.M611186200

[32] Ploner C, Kofler R, Villunger A (2008) Noxa: at the tip of the balance between life and death. Oncogene 27: S84–S92. 19641509 10.1038/onc.2009.46 PMC3272398

[33] Yin X, Giap C, Lazo JS, Prochownik EV (2003) Low molecular weight inhibitors of Myc-Max interaction and function. Oncogene 22: 6151–6159. 13679853 10.1038/sj.onc.1206641

[34] Tsao MS, Sakurada A, Cutz JC, Zhu CQ, Kamel-Reid S, et al (2005) Erlotinib in Lung Cancer – Molecular and Clinical Predictors of Outcome. N Engl J Med 353: 133–144. 16014883 10.1056/NEJMoa050736

[35] Paez JG, Janne PA, Lee JC, Tracy S, Greulich H, et al (2004) EGFR mutations in lung cancer: correlation with clinical response to gefitinib therapy. Science 304: 1497–1500. 15118125 10.1126/science.1099314

[36] Zhou W, Ercan D, Chen L, Yun CH, Li D, et al (2009) Novel mutant-selective EGFR kinase inhibitors against EGFR T790M. Nature 462: 1070–1074 10.1038/nature08622. 20033049 10.1038/nature08622 PMC2879581

[37] Wu X, Li M, Tang W, Zheng Y, Lian J, et al (2011) Design, Synthesis, and In vitro Antitumor Activity Evaluation of Novel 4-pyrrylamino Quinazoline Derivatives. Chem Biol Drug Design 78: 932–940 10.1111/j.1747-0285.2011.01234.x. 10.1111/j.1747-0285.2011.01234.x PMC3412131 21895983

[38] Lessene G, Czabotar PE, Colman PM (2008) BCL-2 family antagonists for cancer therapy. Nat Rev Drug Discov 7: 989–1000 10.1038/nrd2658. 19043450 10.1038/nrd2658

[39] Llambi F, Green DR (2011) Apoptosis and oncogenesis: give and take in the BCL-2 family. Curr Opin Genet Dev 21: 12–20 doi: 10.1016/j.gde.2010.12.001 21236661 10.1016/j.gde.2010.12.001 PMC3040981

[40] Hauck P, Chao BH, Litz J, Krystal GW (2009) Alterations in the Noxa/Mcl-1 axis determine sensitivity of small cell lung cancer to the BH3 mimetic ABT-737. Mol Cancer Ther 8: 883–892. 19372561 10.1158/1535-7163.MCT-08-1118

[41] Lin CL, Tseng HC, Chen WP, Su MJ, Fang KM, et al (2011) Intracellular zinc release-activated ERK-dependent GSK-3[beta]-p53 and Noxa-Mcl-1 signaling are both involved in cardiac ischemic-reperfusion injury. Cell Death Differ 18: 1651–1663. 21660051 10.1038/cdd.2011.80 PMC3172121

[42] Dang CV (1999) c-Myc Target Genes Involved in Cell Growth, Apoptosis, and Metabolism. Mol Cellular Biol 19: 1–11. 9858526 10.1128/mcb.19.1.1 PMC83860

[43] Hoffman B, Liebermann DA (2008) Apoptotic signaling by c-MYC. Oncogene 27: 6462–6472. 18955973 10.1038/onc.2008.312

[44] Tong JS, Zhang QH, Huang X, Fu XQ, Qi ST, et al (2011) Icaritin Causes Sustained ERK1/2 Activation and Induces Apoptosis in Human Endometrial Cancer Cells. PLoS ONE 6: e16781 doi:10.1371/journal.pone.0016781 21408143 10.1371/journal.pone.0016781 PMC3050810

[45] Mei Y, Xie C, Xie W, Tian X, Li M, et al (2007) Noxa/Mcl-1 balance regulates susceptibility of cells to camptothecin-induced apoptosis. Neoplasia 9: 871–881. 17971907 10.1593/neo.07589 PMC2040214

[46] Liu Y, Chen XQ, Liang HX, Zhang FX, Zhang B, et al (2011) Small Compound 6-*O*-Angeloylplenolin Induces Mitotic Arrest and Exhibits Therapeutic Potentials in Multiple Myeloma. PLoS ONE 6: e21930. 21755010 10.1371/journal.pone.0021930 PMC3130785

